# Notes of an European Tour

**Published:** 1846-08

**Authors:** F. H. Hamilton

**Affiliations:** Professor of Surgery in Geneva Medical College.


ART. IV.—*Notes of an European Tour, by* F. H. HAMILTON, M. D. *Professor of Surgery in Geneva Medical College.*

Rome, Hotel D'Allemagne, via Condotti, Italy.

Having secured a vetturino to Rome, in company with my friend Mr. Burchard, and an Italian priest, we came by easy stages through Capua, and across the ancient Liris, traversing the marshes where Marius delivered himself up to Sylla—through the beautiful town of Gaeta, founded by Aneas, it is said, in honor of his nurse Gajeta, a little beyond which is a monument or tower beside the road, marking the spot where Cicero was assassinated, 44 years before the Christian era;—from thence we came on through the old and gloomy town of Fondi, celebrated for the attempt here made by a Turkish fleet to convey off the beautiful Julia, countess of Fondi, but more celebrated forever in my memory for its filth, and its banditti-looking inhabitants. It was on the evening of the second day of our journey that we reached Terracina on the borders of the Pontine Marshes. Alone I climbed the lofty rock which hangs over the modern town, whose summit is crested with the massive ruins of the palace of Theodoric, King of the Ostrogoths; and from this elevated spot I looked over upon one of the most picturesque and extensive views which are found between Naples and Rome.

Four hours after sunrise, the same hour at which Horace left Terracina, when on his road to Brundusium, we commenced our passage across the Pontine Marshes, which extend from here to Forum Appii, a distance of 24 miles, and vary in breadth from 6 to 12 miles. I am thus particular as to the hour because at the time I regarded it as no ordinary event in my life; that we journeyed along the ancient via Appia, which Paul had trod on his way to Rome, and every stage of which Horace has rendered so familiar by his famous itinerery, was alone sufficient to awaken a lively interest in all that I saw; but now I had reached the margin and was about to cross that fearful marsh, whose fatal miasmata had from childhood been associated with the fabled breath of the Upas in the sterile valley of Celebes. Says Pliny "Ob putridas exhalationes harum paludum, ventum Syrophænicum Romæ summopere noxium volunt nonnulli." Yet Rome was 40 miles distant? Mrs. Starke, an English traveller, in her advice to the tourist, remarks, "the inclination to sleep which almost every traveller feels, while breathing this air, should be strenuously resisted; and persons compelled to cross, previous to sunrise, or just before sunset, should be provided with a little strong punch, or powerful wine, and drink it on approaching this district." When therefore I descended first into the plain, I felt somewhat as I have imagined a poor criminal might feel when starting to run the "gauntlet." Nor were my apprehensions a whit abated by the conduct of my companions, both of whom had crossed the marshes before, and whose fears were therefore entitled to respect, even though they had, as they confessed, never felt harm from the exposure. The priest ordered a bottle of wine and the German replenished his flask of "eau de vie"; and although from the season and the sultry air of the morning we anticipated a scorching ride, their heavy overcoats were brought out and wrapped closely about them as if preparing for a Siberian drift. Above all to add to my perplexity and dismay, now for the first I was made acquainted with the story of the Fra Diavoli (Brother Devils), whose bold and frequent attacks upon travellers had not long since rendered these marshes almost impassible except to well armed and considerable bodies. I knew, indeed, that these famous robbers had been at length taken and executed, but at the same time also, most of the Papal soldiers, who had formerly convoyed travellers from post to post, had been withdrawn, and who could tell me that other Italian bandits were not even now looking out from some broken tower upon the neighboring Volscian hills, as a falcon, certain of its feeble quarry?

You have no doubt, often, as certainly I have, pictured to yourself the appearance of this road — a rough broken, "corderoy," cross-logs resting upon the wet, boggy soil, properly varied with ruts, breaks and "dypthongs" —from which far away in each direction stretches a low, flat, black and stagnant fen, covered with rank sedge and a soft, tangled vegetation, crawling with reptiles, and teeming with vile odors and cold miasmus; with here and there a cabin, and a yellow, skeleton family who manage to live in spite of Death. I have studied the picture well and would have affirmed to its accuracy in line and tint. But both form and color have gradually faded, as nearly all that day we drove along a broad, smooth, dry road, raised several feet above the adjacent flats; which latter, like the Western Prairies are covered generally with broad fields of grass, and dotted with herds of buffaloes or cows. I saw also many fine fields of wheat, and at the "posts" small well cultivated garden spots. The soil, it is true, is generally damp, and occasionally quite wet, yet there were few places in the fields bordering the road where I could not walk without inconvenience.

As early as 310 years B. C., Appius Claudius had built this road, (via Appia) with canals and drains through the marshes, which works, since enlarged and extended by Julius Cæsar, Augustus, Trojan, Theodoric and by several successive Popes, still remain. The canals are frequently wide and navigable, and are made to intersect each other in every direction and to enter the sea at several points; in their course they have always sufficient declination to carry slowly the waters which constantly flow from the neighboring mountains into the sea, and also to drain quite effectually the low lands.

As to the "Posts," the two or three which I noticed are comfortable stone buildings, not very clean without, it is true, nor tenanted by pinks of neatness within, yet I confess I have looked in vain among them for those emaciated, jaundiced, and dropsical beings which many travellers pretend abound at Terracina and along this road. We lunched at Bocca di Fiume, the "half-way-house," remaining there from 11 A. M. to 3 P. M., during most of which time I sauntered about in the fields, picking the flowers which cover these plains in such profusion, and especially the red poppy, which is so abundant as to give many fields a beautiful and uniform red color when seen from a distance; and I satisfied myself that the strong odor, of which most travellers speak as arising from decaying vegetation, is only the fragrance of these poppy fields — whether this is sufficient also to produce that inclination to sleep against which Mad. Starke so earnestly cautions us, I cannot positively say, but I do not believe it is. I question, in fact, whether the inclination exists here any more than upon any other rout equally smooth and monotonous.

In short, the Pontine Marshes are doubtless the sources of miasm and miasmatic fevers, probably at nearly all seasons of the year, but no more so than very many of the marshes in our own country in equally warm latitudes, and which, with us who are accustomed to their effects, are not deemed dangerous to pass. They, whose descriptions of the Pontine Marshes have reached us, were generally English travellers, unaccustomed to miasmatic fevers, and their statements of its pestiferous and fatal influence must be received with many grains of allowance.

Near the northern margin of the marshes we stopped to water and refresh our horses at the Post, called by St. Paul "Appii-forum and the Three Taverns," where he was met by the brethren from Rome.

We slept at Velletri, once adorned with the villas of Octavian Augustus, Tiberius, Nerva, Caligula, and Otho; and until long past midnight I sat upon the balcony of my cubiculum, musing. Here dwelt the tyrants of Rome — then, these streets were not as peaceful as now, nor yonder ruins as noiseless — but those hills were the same and the same moon shone upon them, and the same soft air breathed from the Tyrrhean sea — a man cannot live long, nor his palaces — God and Nature alone endure.

Albano is a small town at the foot of Mt. Albano, and 14 miles from Rome. We procured donkeys and guides of an old woman who kept a "livery stable," and leaving our vetturino and dinner at the post we rode up the mountain: and such a noisy hilter-skilter ride, you who have never rode a donkey, with a rope for a bridle and an Italian cicerone attached to his tail with a long stick, as a driver, can never understand or sufficiently admire. My donkey was, I believe, altogether the most asinal of the three, and resolved at length no longer to endure the pulling and punching, and shouting which incessantly annoyed his rear, he gave two or three most accomplished kicks, with some curious ziz-zag flexions which came near bringing both saddle and rider to the ground, and then without waiting to see either adjusted, he scampered away up the hill at a rate neither safe nor agreeable. I think I never shall forget that ride, nor shall I ever forgive my friend Burchard, whose broad dutch laugh seemed to encourage the brute to increase his speed at every step, and not a little, I think, increased the mortal pain which my body suffered at every jump.

But when we had all gathered upon the top of the mountain I believe the ride was soon forgotten. Roma! Roma! exclaimed the guide. Before us lay the Campagna, broad and sterile as a desert: in the midst of which desolation arose the dome of St. Peters, with the towers and obelisks, and long, broken lines of imperial aqueducts reaching across the plain towards the distant hills. Between us and the Mediterranean, a distance of fifteen or twenty miles, lay the scenes of most of the early history of Rome and of all of the last six books of Virgil's Ænead. Along these slopes, amid the tall groves of ilex and oak, through which our road leads us to Rome, the great Diana reared her "peaceful altars" and held her sports, and here moved with pity for slaughtered Virbius, she applied to his mangled corpse those healing herbs, which inspired him with vital breath again. Which act so incensed Jove, that he

> "Struck to the center, with his flaming dart,
> The unhappy founder of the godlike art."

Within sight are Laurentium, Arden "The Proud," Lanuvium, and Alba Longa, all well known to the classical scholar, and upon this very mount stood the jealous Juno, while the Trojans fought their last battle in the plains below. * * * * * *

We are now at length in the "eternal city," where I shall remain until "time" presses me again on the way.

In 1838 the population of Rome was 148,903, of which nearly 5000 were Ecclesiastics and 4000 Jews. There are eight principal hospitals, which together can accomodate about 4000 patients. There are also 13 societies for endowing young girls who are willing to marry! and such also as will dispose of themselves by taking the veil! One would almost believe that at Rome the girl market was overstocked and that these means were necessary to remove the excess. If so, His Holiness might open a very profitable commerce with Texas. About $32,000 per year are said to be distributed in this way, and more than a million of dollars are annually expended by the various charities, including the sums which are sent by public authority to the houses of the poor, not less than 300,000 of which comes from the treasury of the Pope. In addition to all this, large amounts of both money and provisions are daily distributed from the doors of convents within the city. Thus with a population less than half of that in the city of New-York, the amount of charities yearly bestowed is probably ten times as great; yet the number of beggars and lepers which crawl through the streets from morning to night is equal to that of any city in Europe. I have said that the number of Ecclesiastics in the Holy City was, in 1838, nearly 5000. I may add that the number of Foundlings received annually in the various Foundling Hospitals, is about 3000.

La Consolazione, in the rear of the Capitol and under the Tarpeian rock, I visited in company with Mr. Brown the celebrated American Sculptor. It is called the principal Surgical Hospital and is generally pretty well supplied with punctured wounds: we saw none, however, under treatment, and indeed it contained scarcely any cases of interest. The straight splints with straw junks were applied to four or five cases of fractured legs, one poor fellow had a fractured skull, which was dressed without shaving the hair or any other particular regard to cleanliness. Upon one of the beds sat a boy with a scrofulous knee, and one of the finest Roman heads and faces I have ever seen, resembling, I remarked to Mr. Brown, the beautiful antique bust of Young Augustus in the Vatican. Mr. B. who is an enthusiast in his profession, persuaded him to visit his studio as soon as he was able, that he might copy his head. The present number of patients in La Consolazione is about 150; all, or nearly all of whom are placed in one long salle. The situation of this hospital is near the Forum, in the valley between the Capitoline and Palatine Hills, once a marsh, but now within the thronged portion of the modern city; it is therefore not favorably situated for air or cleanliness.

On the way to the Fever Hospital or the Hospital S. Giovanni, we passed through the Forum and among the numerous temples, palaces, triumphal arches, the amphitheatre and other collossal ruins which distinguish this portion of the ancient city, one humble spot, more than all others will remain for ever sacred in my memory; and to this spot alone did my feet repeatedly turn, as more than once I attempted to follow my guide and interest myself in the more stately ruins about me. You may smile at my peculiar romance, but if ever you visit Rome, go along the Forum to where stands upon the Via Sacra the ruins of the celebrated temple of Concord. Close by, in the days of Commodus, stood the drug shop of old Galen, for so he tells us himself. You will not see his sign, nor the illuminated colored bottles at the windows, for the shop was destroyed in a great conflagration, at the same time with the temple, but if you remember well the life of this excellent father and skillful physician, you will turn to mark the place and perhaps linger thereabouts for a while musing, and thinking possibly the old man will come to look after his broken bottles and his plasters and ointments which he prized so highly.

For to judge from the amount of broken walls and loose stones laying about, the fire may have occurred but yesterday.

Galen had enjoyed when he came to Rome in the year 165 the rare privilege of examining a couple of human skeletons at Alexandria, and furnished thus with an extraordinary knowledge of anatomy, he gave public lectures upon the subject to the citizens of Rome. He had thereby attained great celebrity both as a physician and surgeon, when the jealousy, of rival physicians procured his banishment from the city, to which he did not return until recalled by Aurelius, after whose death he was made physician to the young and profligate Emperor Commodus. He lived long afterwards to enjoy his restoration to fame and fortune, having died at the advanced age of 70 years.

